# Phosphorylation and degradation of αB-crystallin during enterovirus infection facilitates viral replication and induces viral pathogenesis

**DOI:** 10.18632/oncotarget.20366

**Published:** 2017-08-19

**Authors:** Gabriel Fung, Jerry Wong, Feaven Berhe, Yasir Mohamud, Yuan Chao Xue, Honglin Luo

**Affiliations:** ^1^ Centre for Heart Lung Innovation, St. Paul’s Hospital and Department of Pathology and Laboratory Medicine, University of British Columbia, Vancouver, BC, Canada; ^2^ Laboratory of Gene Regulation and Signal Transduction, Department of Pharmacology and Pathology, School of Medicine, University of California San Diego, La Jolla, CA, USA

**Keywords:** alphaB-crystallin, viral myocarditis, dilated cardiomyopathy, coxsackievirus, desmin

## Abstract

Protein quality control (PQC) plays a key role in maintaining cardiomyocyte function and homeostasis, and malfunction in PQC is implicated in various forms of heart diseases. Molecular chaperones serve as the primary checkpoint for PQC; however, their roles in the pathogenesis of viral myocarditis, an inflammation of the myocardium caused by viral infection, are largely unknown. AlphaB-crystallin (CryAB) is the most abundant chaperone protein in the heart. It interacts with desmin and cytoplasmic actin to prevent protein misfolding and aggregation and to help maintain cytoskeletal integrity and cardiac function. Here we showed that coxsackievirus infection induced desminopathy-like phenotype of the myocardium, as characterized by the accumulation of protein aggregates and the disruption of desmin organization. We further demonstrated that CryAB was phosphorylated during early and downregulated at later stages of infection. Moreover, we showed that phosphorylated CryAB had a shorter half-life and was targeted to the ubiquitin-proteasome system for degradation. Lastly, we found that overexpression of CryAB significantly attenuated viral protein production and progeny release, indicating an anti-viral function for CryAB. Together, our results suggest a mechanism by which coxsackieviral infection induces CryAB degradation and loss-of-function, resulting in desmin aggregation, ultimately contributing to compromised cytoskeletal integrity and viral cardiomyopathy.

## INTRODUCTION

Coxsackievirus B3 (CVB3) is a human pathogenic enterovirus in the family *Picornaviridae*. It is amongst the most prevalent etiological agents associated with myocarditis, an inflammation of the myocardium [1]. Viral myocarditis can cause reduced cardiac function and the development of dilated cardiomyopathy (DCM), which may result in sudden death in children or end-stage heart failure in adults [1]. However, the molecular and pathological mechanism of viral myocarditis and its progression to DCM has not yet been fully understood.

Protein quality control (PQC), achieved through the function of molecular chaperones and protein degradation systems, is an essential mechanism in maintaining cellular homeostasis [2, 3]. Molecular chaperones serve as the first line of defense for PQC by assisting proper protein folding, and through protecting proteins against stress-induced misfolding and aggregation. The protein degradation systems, including the ubiquitin-proteasome pathway and autophagy, remove terminally misfolded or damaged proteins. Dysfunction in PQC has been implicated in the development of heart diseases [3–6]. We have previously demonstrated that CVB3 infection disrupts the function of cellular protein degradation pathway, resulting in aberrant accumulation of ubiquitin conjugates [7–10]. However, the role of molecular chaperones in the pathogenesis of enteroviral myocarditis and DCM remains to be defined.

AlphaB-crystallin (CryAB) is the most abundant chaperone protein in the myocardium and belongs to a family of small heat shock proteins (sHSP) [11, 12]. CryAB interacts with intermediate filament protein desmin and cytoplasmic actin to prevent protein misfolding and aggregation and to ensure cytoskeletal integrity and proper cardiomyocyte function [13, 14]. There is increasing evidence that CryAB acts as an important regulator of cytoprotection against various forms of cellular stress in the heart. Overexpression of CryAB in cultured cardiomyocytes and in transgenic mouse hearts protects against ischemia/reperfusion injury [15, 16] and attenuates cardiac hypertrophy elicited by pressure-overload [17]. Double knockout of CryAB and HSPB2, a CryAB-related sHSP, in mouse heart induces abnormal cardiac growth and defective myocardial relaxation [18, 19]. It has also been shown that cardiac-specific expression of CryAB^R120G^, a mismatch mutant genetically associated with human desmin-related cardiomyopathy (also known as desminopathy) [20], results in abnormal desmin aggregation and heart failure in mice [14, 21]. Impaired mitochondrial function [22, 23] and reductive stress [24] have been identified as key cellular mechanisms leading to the pathogenesis of desminopathy.

In this study, we investigated the regulation and functional role of CryAB in CVB infection and viral myocarditis. We showed that CVB3 infection causes a pathological phenotype similar to desmin-related cardiomyopathy. We further demonstrated that CVB3 infection leads to early phosphorylation and late degradation of CryAB in a proteasome-dependent manner. Finally, our data revealed an anti-viral function for CryAB against CVB3 infection. Collectively, our findings in this study suggest a novel desminopathy-like mechanism of viral myocarditis and its progression to DCM.

## RESULTS

### Coxsackievirus infection induces accumulation of pre-amyloid oligomers

**Figure 1:**
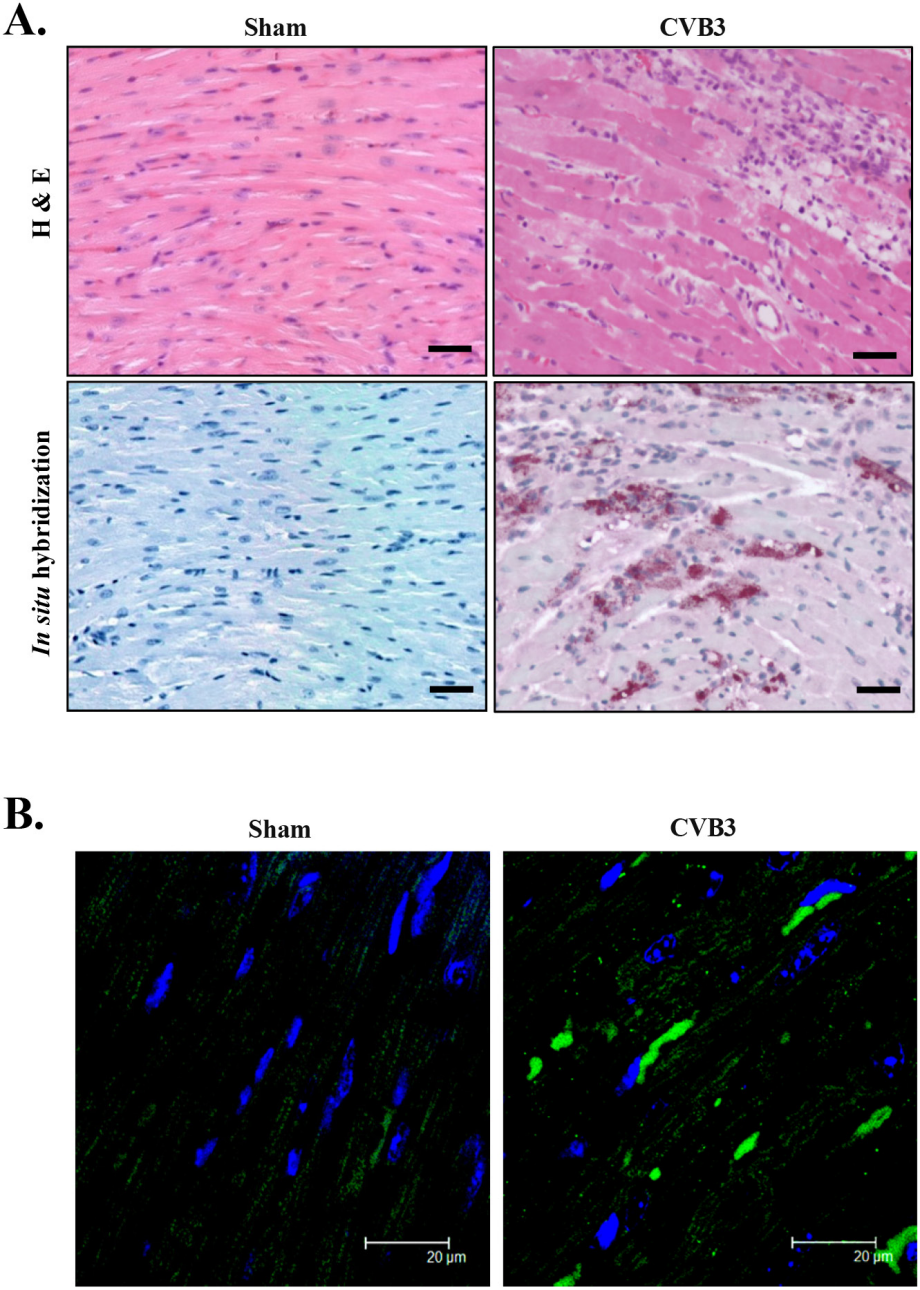
CVB3 infection causes accumulation of pre-amyloid oligomers in virus-infected mouse heart A/J mice were sham- or CVB3-infected for 9 days and mouse hearts were harvested. **(A)** H&E staining and *in situ* hybridization were performed to assess myocardial injury and viral genomic RNA (red). The nucleus was counterstained with DAPI (blue). Bar=30 μm. **(B)** Immunofluorescent staining was performed to detect pre-amyloid oligomers (green).

Mounting evidence suggests that cardiomyopathies share a similar protein conformational pathology as neurodegenerative diseases [4, 5]; however the presence of toxic protein aggregates in virus-induced cardiomyopathy remains uncharacterized. Pre-amyloid oligomers (PAOs) are a form of protein aggregation that was shown to be toxic to cardiomyocytes [25]. Here we performed immunofluorescent staining to determine whether PAOs are present in virus-infected heart. A/J mice, a mouse strain susceptible to CVB3-induced myocarditis/cardiomyopathy, were infected with CVB3 for 9 days. Histological examination and *in situ* hybridization were conducted on mouse heart to assess virus-induced injuries and viral genomic RNA, respectively. Hematoxylin & eosin (H&E) staining showed myocardial damage in CVB3-infected heart, associated with mononuclear lymphocyte infiltration, a defining pathology of myocarditis (Figure 1A, top panel). *In situ* hybridization further confirmed the existence of viral transcripts in virus-infected heart (Figure 1A, bottom panel). Finally, immunofluorescent staining demonstrated an increased accumulation of PAOs, as observed by large immunoreactive perinuclear aggregates (Figure 1B), suggesting that protein homeostasis is disrupted in the virus-infected heart, leading to aberrant accumulation of protein aggregates.

### Coxsackievirus infection causes the accumulation and structural disruption of desmin

**Figure 2:**
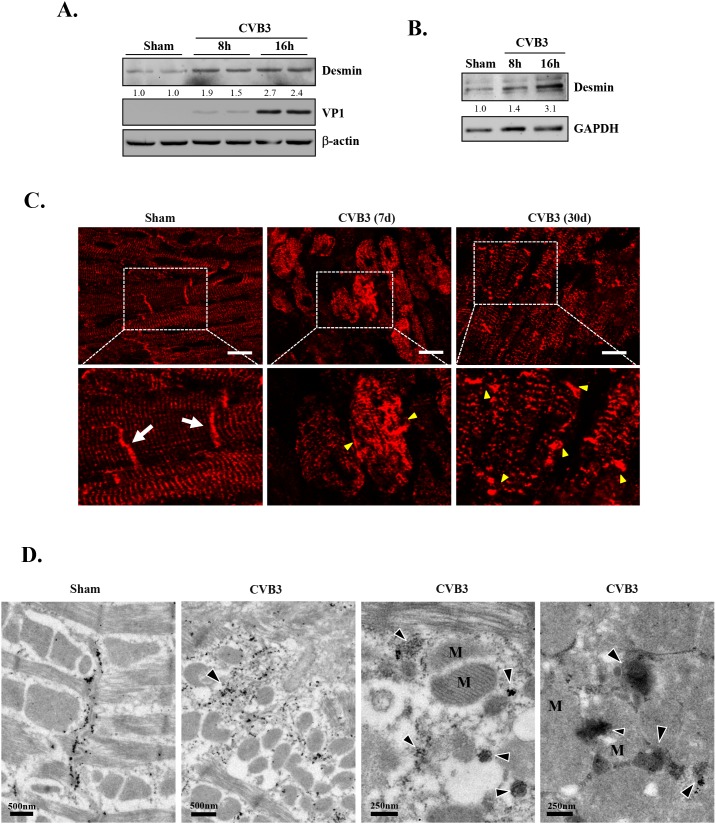
CVB3 infection leads to increased protein levels of desmin *in vitro* and results in the disruption of desmin structures *in vivo* HL-1 murine cardiomyocytes **(A)** and rat primary neonatal cardiomyocytes **(B)** were sham- or CVB3-infected for 8 or 16 hours as indicated. Cell lysates were collected for western blot analysis of desmin and β-actin/GAPDH (loading control). **(C)** A/J mice were sham- or CVB3-infected for 7 or 30 days as indicated. Mouse hearts were harvested and immunofluorescent staining for desmin was carried out (red). Note that desmin in sham-infected mouse hearts is predominantly located on the Z lines and intercalated discs (white arrows). After CVB3 infection, the striated pattern of desmin is disrupted accompanied by the presence of granular-like aggregates (yellow arrowheads). Bar=10 μm. **(D)** A/J mice were sham- or CVB3-infected for 9 days and mouse hearts were harvested for immuno-electron microscopic study of desmin. Representative images are presented. Note the redistribution of desmin from the intercalated discs in the sham to the cytosolic electron dense aggregates (arrowheads) in CVB3-infected heart, as well as the disruption of mitochondrial structure and striated patterns of sarcomeres. M, mitochondria.

Although the exact components of PAOs in the heart are still not clear, desmin aggregates have been reported to be the major PAO element in desmin-related cardiomyopathy [26]. To explore whether the CVB3-infected heart also exhibits abnormal accumulation of desmin, we examined protein expression and distribution of desmin in virus-infected cardiomyocytes and mouse hearts. Western blot results showed that protein levels of desmin were increased in CVB3-infected HL-1 mouse cardiomyocytes (Figure 2A) and rat primary neonatal cardiomyocytes (Figure 2B). Immunohistochemistry revealed that desmin in sham-infected mouse hearts was predominantly located on the Z lines and intercalated discs (Figure 2C, white arrows). However, after CVB3 infection, the striated pattern of desmin was disrupted and changed to granular-like aggregates (Figure 2C, yellow arrowheads). In addition, the characteristic localization of desmin at the intercalated discs found in the sham group was indistinct or absent in CVB3-infected hearts (Figure 2C). Desmin disorganization was observed at different virus-infected areas across the whole section. Finally, immuno-gold labeling for desmin confirmed the presence of desmin-positive, electron dense aggregates (arrowheads) in CVB3-infected hearts (Figure 2D). Figure 2D also showed the disruption of striated patterns of sarcomeres and the compromised mitochondrial structure in virus-infected hearts. Collectively, our results suggest that CVB3 infection causes a pathological phenotype similar to desmin-related cardiomyopathy.

### Coxsackievirus infection results in early phosphorylation and late downregulation of CryAB

**Figure 3:**
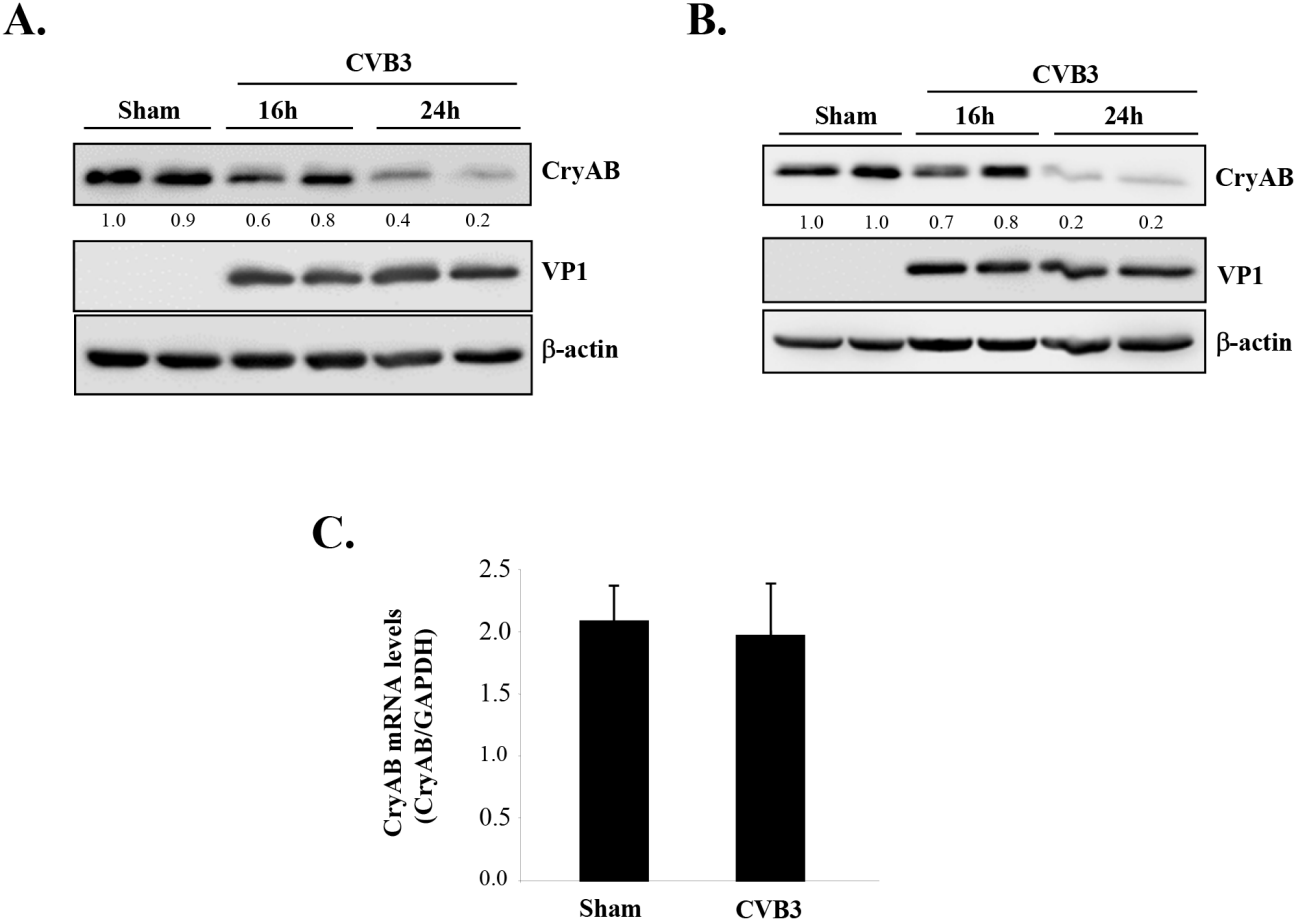
CVB3 infection leads to downregulation of CryAB HL-1 murine cardiomyocytes **(A)** and rat primary neonatal cardiomyocytes **(B)** were infected with CVB3 for 16 or 24 hours as indicated. Cell lysates were collected for Western blot analysis of CryAB and β-actin (loading control). Protein levels of CryAB were quantitated by densitometric analysis using NIH ImageJ, normalized to β-actin, and presented as fold changes compared with sham-infected sample, which was arbitrarily set a value of 1.0. Relative protein levels are listed underneath each blot. **(C)** RNA was extracted from primary rat neonatal cardiomyocytes infected with CVB3 for 24 hours. Gene levels of CryAB were measured by real-time quantitative PCR using the TaqMan technology and normalized to *GAPDH* mRNA (mean ± SD, n=3). The difference between sham and CVB3 group is not statistically significant.

As CryAB is the primary molecular chaperone in the heart assisting correct assembly and proper folding of desmin [14, 27], we questioned whether CryAB is dysregulated during CVB3 infection, contributing to viral cardiomyopathy. To address this, we examined protein and gene levels as well as phosphorylation status of CryAB. As shown in Figure 3A & 3B, we demonstrated that protein levels of CryAB were markedly decreased in the late stage of viral infection (i.e. 24 hours post-infection) in HL-1 mouse cardiomyocytes (Figure 3A) and rat neonatal cardiomyocytes (Figure 3B).

The *CryAB* has been identified as a novel target gene of tumor suppressor protein p53 that directly transactivates *CryAB* and induces its gene and protein expression [28]. We have previously shown that CVB3 infection leads to degradation of p53 [29], raising the possibility that CVB3 may downregulate *CryAB via* reducing the levels of p53. To determine whether *CryAB* is transcriptionally downregulated during CVB3 infection, we conducted real-time qRT-PCR and demonstrated that mRNA levels of *CryAB* were unaltered following infection (Figure 3C), suggesting that the reduced protein expression of CryAB is unlikely a result of decreased gene expression.

**Figure 4:**
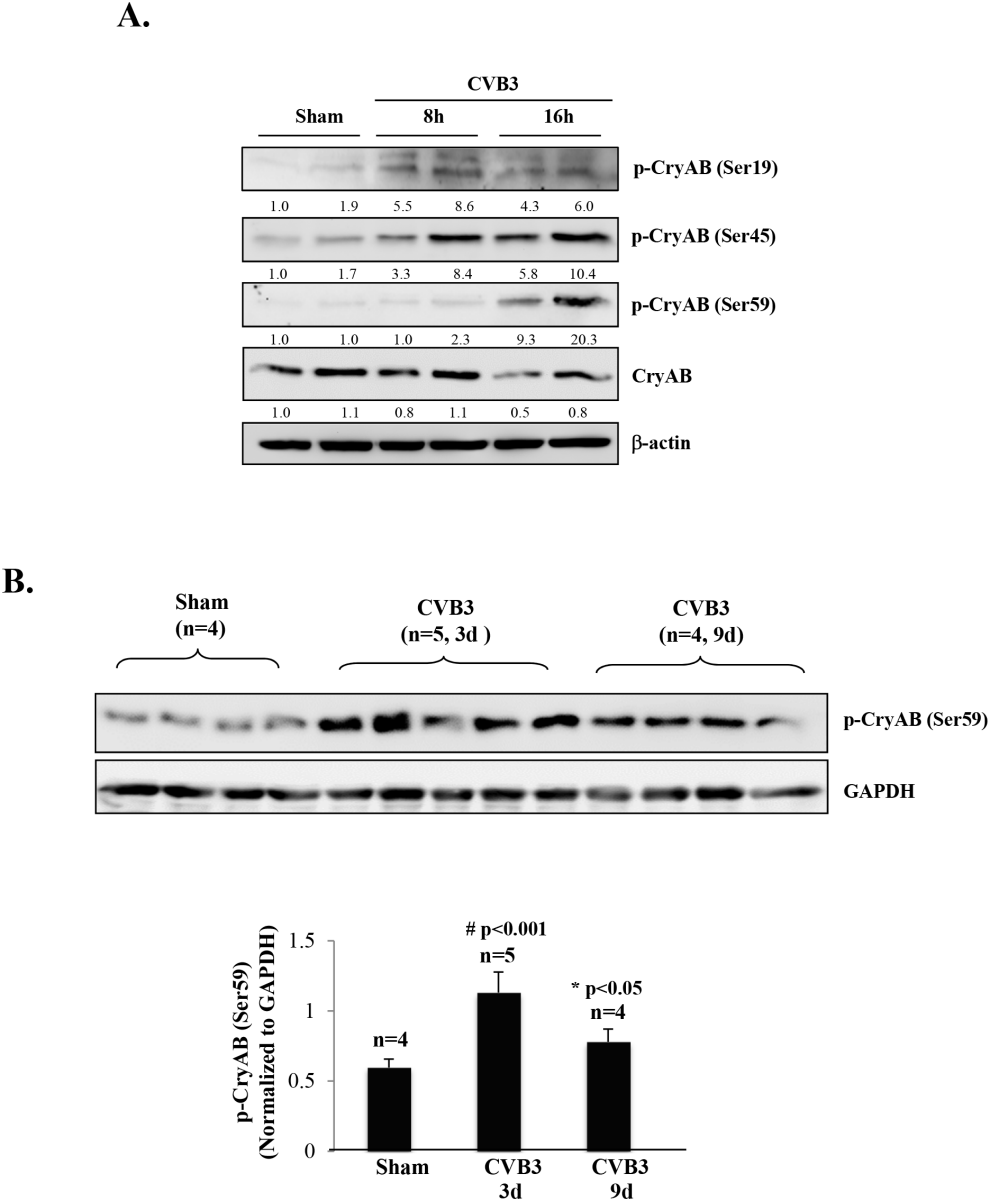
CVB3 infection results in phosphorylation of CryAB **(A)** HL-1 mouse cardiomyocytes were sham-infected or infected with CVB3 for 8 or 16 hours as indicated. **(B)** A/J mice were sham- or CVB3-infected for 3 or 9 days as indicated. Cell or tissue lysates were collected for Western blot analysis of phospho-CryAB (p-CryAB), CryAB, and β-actin/GAPDH (loading control). Protein levels of p-CryAB were quantitated by densitometric analysis, normalized to β-actin or GAPDH, and presented as fold changes compared with sham (which was arbitrarily set a value of 1.0) in (A) or as mean ± SD in (B) lower panel.

Moreover, we examined phosphorylation status of CryAB upon CVB3 infection. In response to cellular stress, it has been reported that CryAB can be phosphorylated on three serine residues (i.e. ser-19, ser-45, and ser-59) [30, 31]. We showed that viral infection resulted in an increased phosphorylation of CryAB on all three serine residues at 8 and 16 hours post-infection (Figure 4A). Western blot analysis of CVB3-infected mouse heart also revealed enhanced phosphorylation of CryAB at day 3 and 9 post-infection (Figure 4B).

### Phosphorylated CryAB is targeted to the ubiquitin-proteasome pathway for degradation

**Figure 5:**
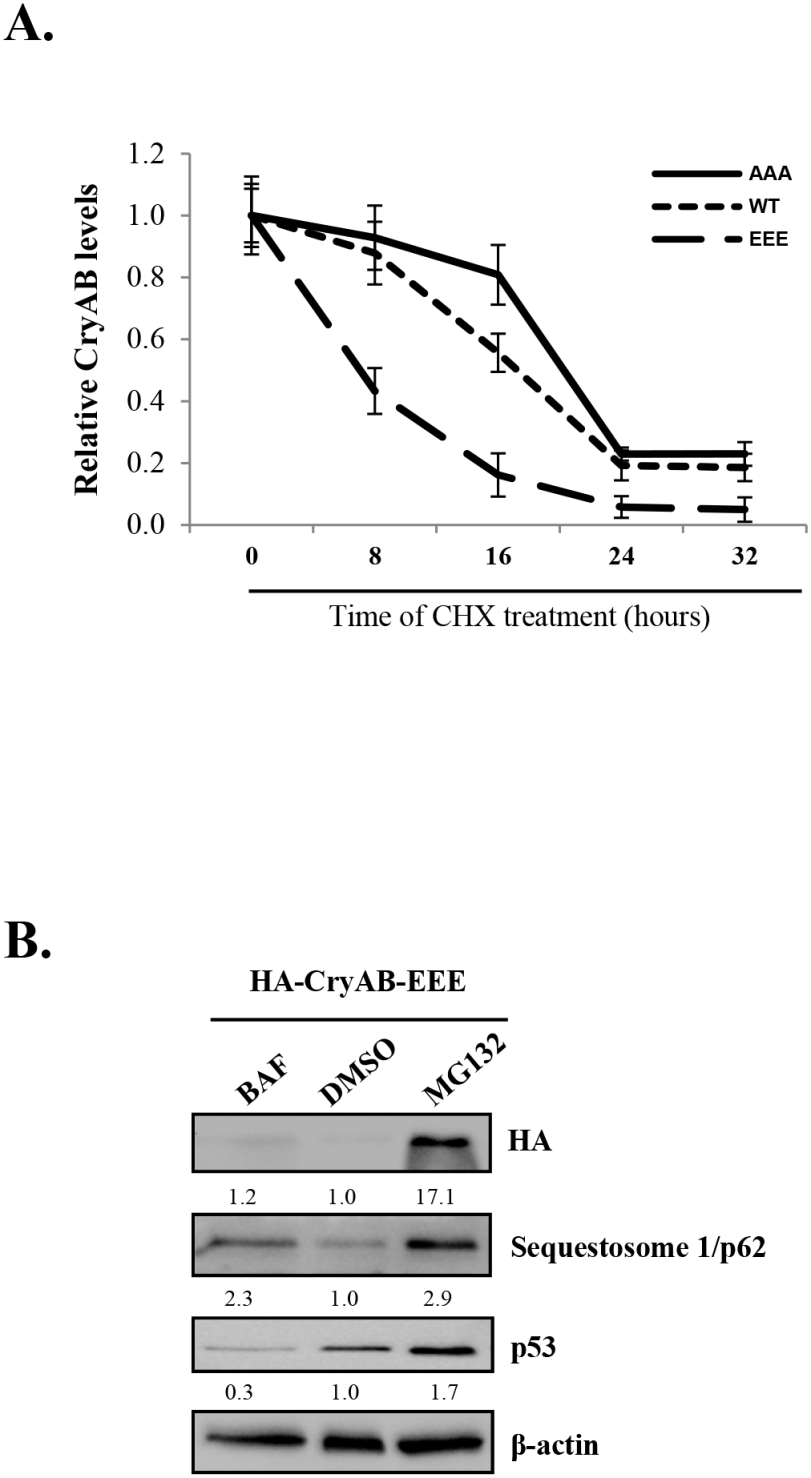
Phosphorylated CryAB exhibits reduced stability **(A)** HL-1 mouse cardiomyocyteswere transduced with adenoviral HA-CryAB-WT, -AAA, or -EEE as indicated, followed by treatment with cycloheximide (CHX, 20μg/ml) for 0, 8, 16, 24 and 32 hours. Western blot analysis was performed and CryAB expression was quantitated by densitometric analysis, normalized to b-actin, and presented as fold changes compared with cells treated with cycloheximide at 0 hour, which was arbitrarily set a value of 1.0 (mean ± SD, n=3). **(B)** HL-1 cells transduced with HA-CryAB-EEE were treated with 20 nM bafilomycin A1 (BAF), vehicle (DMSO), or 4.0 μM MG132 for 48 hours and Western blot analysis was conducted for detection of HA-CryAB, p62, p53, and β-actin (loading control). Densitometric analysis was conducted as above and data are presented below each blot as fold changes compared with DMSO-treated control.

Post-translational modification by phosphorylation has a variety of functions, including the regulation of protein degradation. Phosphorylation of some proteins, such as IκBα, has been shown to be required for ubiquitin ligase recognition and consequent substrate ubiquitination and proteasome degradation [32]. To understand the significance of CryAB phosphorylation in its stability, we utilized three adenoviral constructs expressing phosphomimetic mutant (HA-CryAB-EEE), non-phosphorylatable mutant (HA-CryAB-AAA), and wild-type of CryAB (HA-CryAB-WT), respectively. We conducted cycloheximide pulse-chase assay to determine the effects of CryAB phosphorylation on its turnover. HL-1 cells transduced with various CryAB constructs were treated with cycloheximide, a protein synthesis inhibitor, for 0, 8, 16, 24, and 32 hours. CryAB protein expression was examined by Western blotting. As shown in Figure 5A, CryAB-WT protein levels decreased to ∼50% after 16-hour cycloheximide treatment. The turnover rate of phosphomimetic mutant of CryAB (EEE) was notably higher than that of CryAB-WT (lost ∼50% protein in less than 8-hour cycloheximide treatment), whereas non-phosphorylatable CryAB mutant (AAA) displayed a decreased degradation rate (protein level reduced to ∼50% at ∼20-hour treatment of cycloheximide). Our results suggest that phosphorylation of CryAB may contribute to decreased protein stability.

We further investigated the pathways through which phosphorylated CryAB is degraded. Figure 5B showed that, while treatment with bafilomycin A1, a lysosome inhibitor that blocks autophagic degradation, failed to alter protein levels of CryAB, the addition of proteasome inhibitor, MG132, markedly rescued CryAB protein expression, suggesting proteasome-dependent degradation of phosphorylated CryAB. The detection of sequestosome 1/p62 and p53 was used as positive controls for effective blockage of the autophagy and the proteasome pathway by bafilomycin and MG132, respectively (Figure 5B).

### CryAB plays an anti-viral role against coxsackievirus infection

**Figure 6:**
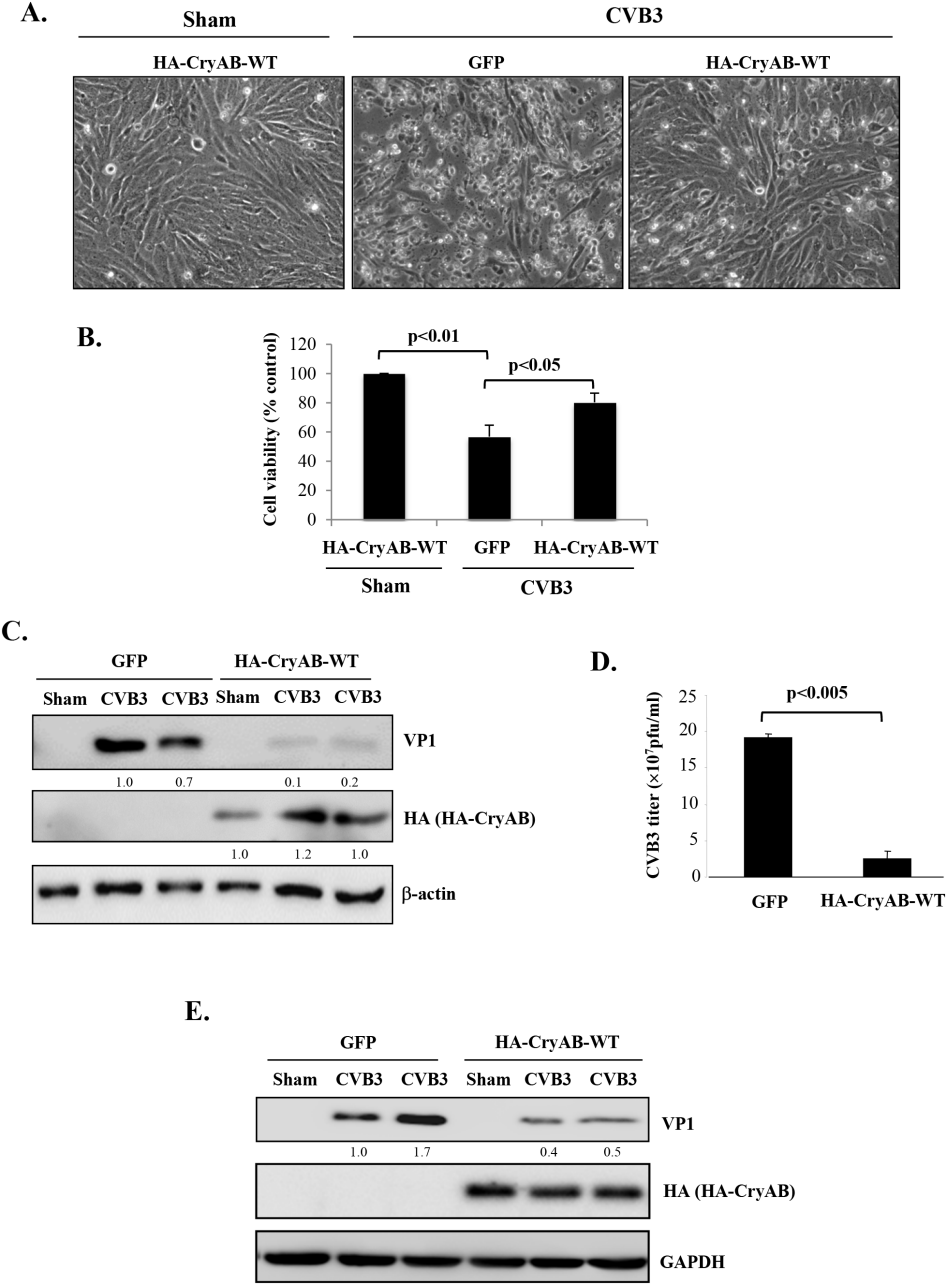
Overexpression of wild-type CryAB reduces CVB3 infection HL-1 mouse cardiomyocytes **(A-D)** and rat neonatal cardiomyocytes **(E)** were transduced with adenoviral HA-CryAB-WT or control vector (GFP) for 48 hours, and then infected with CVB3 for 24 hours. (A) Representative images of sham and CVB3-infected HL-1 cells overexpressing HA-CryAB-WT or GFP control as indicated. (B) Cell viability was determined by the MTS assay and presented as percentage changes as compared with sham infection that was arbitrarily set as 100% (mean ± SD, n=3). (C, E) Western blotting was carried out to examine protein levels of viral capsid protein VP1, HA-tagged CryAB, and β-actin/GAPDH (loading control) in HL-1 (C) and rat neonatal cardiomyocytes (E). VP1 levels were quantitated by densitometric analysis, normalized to β-actin, and presented underneath as fold changes compared with the GFP-transduced, CVB3-infected cells. (D) Plaque assay was performed to measure CVB3 viral progeny titer in supernatants collected from CVB3-infected HL-1 cells transduced with HA-CryAB-WT or GFP control vector (mean ± SD, n=3).

Finally, we studied the role of CryAB in CVB3 infection. HL-1 cardiomyocytes transduced with HA-CryAB-WT or control construct were infected with CVB3 for 24 hours. We demonstrated that the expression of CryAB-WT conferred resistance to viral infection, as evidenced by reduced cytopathic effects (Figure 6A), increased cell viability (Figure 6B), decreased production of viral protein (Figure 6C & 6E), significantly lowered viral titer (∼ 20 fold reduction) (Figure 6D) in CryAB-WT-overexpressing cells. The lack of significant degradation of exogenously expressed HA-CryAB-WT in Figure 6C & 6E is due to two possible reasons: (1) Overexpression of HA-CryAB-WT inhibits viral replication. Thus decreased CryAB degradation is likely a result of reduced viral replication; (2) high expression levels of exogenous HA-CryAB exceed the degradation capacity of proteasome, leading to decreased degradation. Together, our results suggest that CryAB may act as an anti-viral protein against CVB3 and that dysregulation of CryAB may provide a favorable environment for effective viral infection.

## DISCUSSION

The current study addresses a novel function and mechanism of CryAB dysregulation in the pathogenesis of viral myocarditis/cardiomyopathy. Using A/J mice, a mouse strain susceptible to CVB3-induced myocarditis, we observed an abnormal accumulation of pre-amyloid oligomers in the myocardium of virus-infected heart, accompanied by disarrangement of desmin-associated cytoskeletal network. In virus-infected cardiomyocyte cultures, we demonstrated that CVB3 infection induces early phosphorylation and late degradation of CryAB by the proteasome. Lastly, we revealed an anti-viral activity for CryAB against CVB3 infection. Taken together, our findings in this study suggest a novel mechanism of the pathogenesis of viral myocarditis, i.e. CVB3 infection causes loss of host CryAB protein and consequent collapse of the cytoskeletal network and increased viral load, contributing to the development of viral myocarditis and its progression to DCM.

Studies in the past decade have documented the presence of misfolded protein aggregates in various types of cardiac diseases, including cardiac hypertrophy and dilated and ischemic cardiomyopathies [5]. In this study, we demonstrated for the first time that virus-induced myocarditis also displays pathological phenotypes of protein conformational diseases, including abnormal accumulation of peri-nuclear pre-amyloid oligomers and disruption of desmin network, suggesting that protein homeostasis is disrupted upon CVB3 infection of cardiomyocytes.

CryAB is constitutively expressed in the myocardium under normal growth conditions. When the heart undergoes stress, CryAB is considerably induced. Expression of CryAB has been reported to be greatly induced in the cardiac tissue of patients with familial hypertrophic cardiomyopathy [33], desmin-related cardiomyopathy [34], and pressure-overload cardiac hypertrophy [17]. It is proposed that increased CryAB protein expression during cellular stress is a compensatory mechanism to combat an increased burden of protein misfolding. However, in end-stage heart failure, CryAB was found to be downregulated [35]. Our data in this study showing reduced protein levels of CryAB in CVB3-infected cardiomyocytes suggests a late-stage, de-compensatory effect of CryAB during disease progression.

In response to cellular stress, CryAB can be phosphorylated at three serine residues (Ser-19, Ser-45, and Ser-59) [30, 31]. The underlying significance of CryAB phosphorylation remains unclear and available data are controversial. Several studies have suggested that phosphorylation of CryAB in response to ischemia/reperfusion injury may enhance its cytoprotective function by promoting the translocation of CryAB from the cytosol to the cytoskeleton and myofilaments, where it assists in stabilizing these cardiac components [36–38] and preventing mitochondrial damage [39]. However, other studies have indicated that phosphorylated CryAB may be detrimental for myocardial contractile function. Serine phosphorylation of CryAB was reported to reduce oligomerization and chaperone activity of CryAB [30, 40]. Pseudo-phosphorylation of all three serine resides on CryAB abolishes the anti-apoptotic function of CryAB, suggesting that phosphorylation negatively regulates the cytoprotective function of CryAB [41]. Moreover, it was reported that cardioplegia and cardiopulmonary bypass induced CryAB phosphorylation on Ser-45 and Ser-59 and that increased CryAB phosphorylation negatively correlated with cardiac function after surgery [42].

In this study, we characterized the relationship between CryAB phosphorylation and degradation and determined whether phosphorylation of CryAB may affect its stability. Our data showed the phosphorylation of CryAB at serine-19, -45 and -59 upon CVB3 infection. Using phospho-mutants of CryAB, we found that HA-CryAB-AAA mutant yielded significantly higher protein stability as compared to its WT counterpart, particularly at 16 hours after cycloheximide treatment. HA-CryAB-EEE protein level was significantly lower than its WT and non-phosphorylatable controls, with ∼20% protein expression remaining by 16 hours cycloheximide treatment. This data suggests that CryAB phosphorylation at serine-19, -45, and -59 leads to increased CryAB protein degradation, possibly contributing to desmin misfolding and compromised cytoskeletal integrity. We further demonstrated that inhibition of the proteasome activity, but not the lysosome function, successfully restores CryAB protein expression of the - EEE mutant, suggesting a ubiquitin-proteasome-dependent degradation of phosphorylated CryAB. Future investigation is warrant to identify the specific E3 ligase responsible for CryAB ubiquitination/degradation, which would be crucial for understanding viral cardiomyopathy and other CryAB-related proteinopathies.

Our results in this study demonstrate that virus-induced cytopathic effects, viral protein expression and titer decrease after overexpressing CryAB. The exact mechanism by which CryAB reduces CVB3 infectivity is still unclear. It has been previously reported that disruption of the cytoskeletal network is required for efficient propagation and replication of CVB3 [43]. Knockdown of CryAB has been shown to result in abnormal accumulation of desmin aggregates and subsequent collapse of the cytoskeletal network [13, 14, 27]. Thus, it is postulated that impairing the integrity of the cytoskeletal network is an important strategy utilized by CVB3 to facilitate its propagation, and that overexpression of CryAB partially restores the functional network, resulting in reduced viral infection. However, a direct effect of CryAB on CVB3 cannot be completely excluded at the moment.

**Figure 7:**
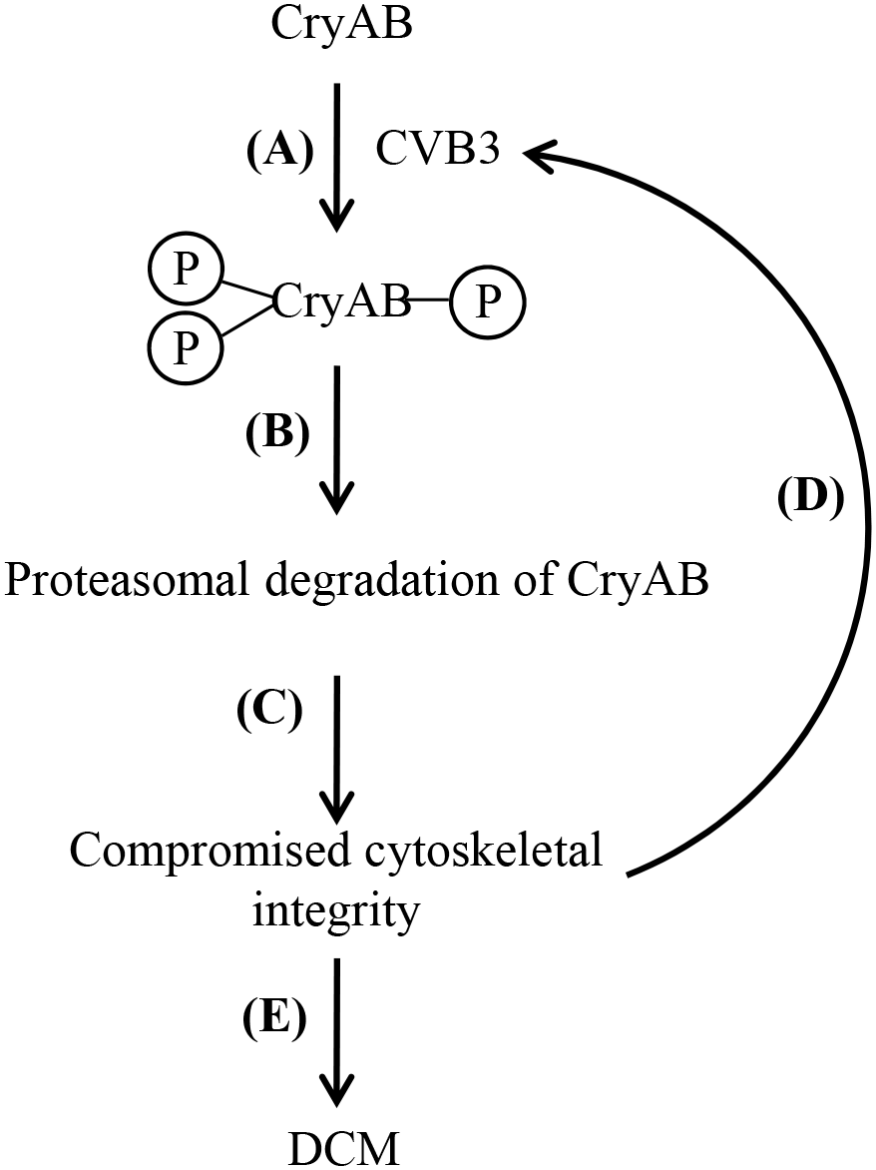
Proposed function and mechanism of CVB3-mediated CryAB dysregulation in the development of viral cardiomyopathy Upon CVB3 infection, CryAB is phosphorylated at residues serine-19, -45 and -59 **(A)**. Phosphorylated CryAB is then targeted for proteasomal degradation **(B)**, which leads to impaired cytoskeletal integrity **(C)**, contributing to increased viral propagation **(D)**, and ultimate DCM **(E)**.

Based on the findings of the current study, we propose a novel mechanism by which CVB3 infection targets CryAB contributing to virus-induced cardiomyopathy. During resting states, cytoskeletal integrity and cardiomyocyte function is maintained partly due to the chaperone activity of CryAB. Upon CVB3 infection, CryAB is phosphorylated at serine-19, -45, and -59 (Figure 7A), followed by the degradation of CryAB *via* the proteasomal pathway (Figure 7B). Loss of CryAB results in compromised integrity of cytoskeletal network (Figure 7C), thereby enhancing viral propagation (Figure 7D) and ultimately leading to the development of DCM (Figure 7E).

In summary, our studies not only provide novel insights into the pathogenic mechanisms of viral myocarditis, but also offer an excellent model system to understand the regulation and function of CryAB in other heart diseases. In addition, the combined beneficial effects of CryAB in defense against various types of cardiac injury and viral infection make it a very attractive drug target for clinical translation. It has been shown that nano-particles encapsulated CryAB-derived mini-peptides, which are designed based on the identified sequences of CryAB exhibiting chaperone activity, offer cytoprotection against oxidative stress and other injuries in human retinal pigment epithelial cells [44]. It is anticipated that a similar strategy can be used to deliver mini-chaperone peptides into the myocardium for the treatment of viral myocarditis and the related DCM. Furthermore, future development of small molecules that restore/enhance the function of CryAB will yield potential therapeutic approaches that are not only useful for treating viral myocarditis, but also other proteinopathy-based diseases.

## MATERIALS AND METHODS

### Cell culture

The HL-1 mouse cardiomyocytes, a generous gift from Dr. William Claycomb at the Louisiana State University Medical Center, were cultured in Claycomb media (Sigma-Aldrich, #51800) supplemented with 10% heat-inactivated newborn fetal bovine serum, 100 μg/ml penicillin-streptomycin, 2 mM L-glutamine, and 0.1 mM norepinephrine as described previously [10].

Rat neonatal cardiomyocytes were obtained by enzymatic dissociation of cardiac ventricles from 1-2 day old Sprague-Dawley rat neonates as previously described [45]. Non-myocytes were removed through two rounds of pre-plating and cytosine 1-β-D-arabinofuranoside (Sigma-Aldrich, #C1768) was added to inhibit the growth of contaminating non-myocytes. The enriched cardiomyocytes were cultured in Dulbecco's modified Eagle’s media with 10% bovine calf serum and 10% horse serum**.**

### Viral infection and treatment in cell culture

Cardiomyocytes were infected with CVB3 (Kandolf strain) at a multiplicity of infection (MOI) of 10 or sham infected with equal volumes of phosphate-buffered saline (PBS) for 1 hour in serum-free media. Cells were then washed with PBS and placed in fresh complete culture media for various time points. For experiments treated with proteasome inhibitor MG132 (Sigma-Aldrich, #C2211) or lysosome inhibitor bafilomycin A1 (LC Laboratories, #B-1080), agents were added to the fresh media after 1-hour viral infection for various periods of times as indicated. For pulse-chase experiment, HL-1 cardiomyocytes were treated with protein synthesis inhibitor, cycloheximide (20μg/ml, Sigma-Aldrich, #C7698), for 0, 8, 16, 24, and 32 hours in the absence of CVB3 infection. Equal volumes of vehicle DMSO were used as a control for all drug treatment studies.

### Mouse infection

CVB3 infection of A/J mice (Jackson Laboratory, #000646), a mouse strain susceptible to CVB3 infection, was performed as previously described [46]. In brief, male mice at age 4-5 weeks were either infected intraperitoneally with 10^5^ plaque-forming units (pfu) of CVB3, or sham-infected with the same volumes of PBS. After the indicated days of infection, mice were killed and hearts were harvested for subsequent analysis. All mouse studies were approved by the Animal Care Committee at the University of British Columbia.

### Adenoviral transduction

Recombinant adenoviruses expressing HA-tagged wild-type (HA-CryAB-WT), phosphomimetic (HA-CryAB-EEE, in which Ser-19, -45, and -59 residues were replaced with glutamic acid to mimic phosphorylation), and non-phosphorylatable CryAB (HA-CryAB-AAA, in which Ser-19, -45, and -59 residues were replaced with alanine to block phosphorylation) were generously provided by Dr. Christopher Glembotski at the San Diego State University. For adenoviral transduction, rat neonatal cardiomyocytes or HL-1 murine cardiomyocytes at 50∼60% confluence were incubated with serum-free media containing recombinant adenovirus (MOI = 50) for 3 hours. After two washes with PBS, the cells were incubated in complete media for various times as indicated. Adenoviral construct expressing GFP was used as a control.

### Western blot analysis

Cells or tissues were harvested in lysis buffer (50 mM NaPyrophosphate, 50 mM NaF, 50 mM NaCl, 5 mM EDTA, 5 mM EGTA, 100 μM Na_3_VO_4_, 10 mM HEPES, and 2% SDS). Western blot analysis was performed as previously described [47]. The primary antibodies used in this study include antibodies against VP1 (DakoCytomation, #M706401-1), desmin (Cell Signaling, #5332), CryAB (StressMarq, #SMC-165), p-Ser19-CryAB (Enzo Life Sciences, #ADI-SPA-225), p-Ser45-CryAB (Enzo Life Sciences, #ADI-SPA-226), p-Ser59-CryAB (Enzo Life Sciences, #ADI-SPA-227), HA (Roche, #11867423001), sequestosome 1/p62 (PROGEN Biotechnik GmbH, #GSQSTM1-C), and p53 (Santa Cruz Biotechnology, #sc-126). The level of protein was quantified by densitometric analysis using NIH ImageJ software, normalized to GAPDH or β-actin, and presented as fold changes compared with non-challenged controls, which were arbitrarily set a value of 1.0.

### Immuno-electron microscopy

Immuno-electron labeling for desmin was performed as previously described [47]. Briefly, rabbit anti-desmin polyclonal antibody (Abcam, #ab39533) was used at 1:50. F(ab') 2 fragment of ultra-small goat-anti-rabbit IgG was used at 1:50. Following steps were performed using a Pelco Biowave Microwave. Controls were incubated in normal goat serum diluted at 1:60. Sections were stained in 2% uranyl acetate and lead citrate, air dried and analyzed on a Tecnai 12 electron microscope.

### Cell viability assay

A modified 3,4-(5-dimethylthiazol-2-yl)-5-(3-carboxymethoxyphenyl)-2-(4-sufophenyl)-2H-tetrazolium salt (MTS) assay (Promega, #G3581) was used to measure cell viability following the manufacturer’s instruction.

### Plaque assay

Virus titer in the cell supernatant was measured by an agar overlay plaque assay as previously described [7]. Plaques were counted and virus titers were calculated and expressed as pfu/ml.

### Immunofluorescent staining

Indirect immunofluorescent staining was performed as previously described [10]. Primary and secondary antibodies used in this study include anti-pre-amyloid oligomers (Millipore, #AB9234), anti-desmin (Cell Signaling, #5332), alexa-fluor-594 goat anti-rabbit IgG (Invitrogen, #A11012), and alexa-fluor-488 goat anti-mouse IgG (Invitrogen, #A11001). Following secondary antibody incubation and PBS wash, coverslips were mounted using DAPI-mounting solution (VectaShield, #H1200).

### Histology and *in situ* hybridization

Mid-portions of sham- and CVB3-infected mouse hearts were formalin-fixed, paraffin-embedded, and then stained with H&E as described previously [48]. *In situ* hybridization was carried out to detect viral RNA as previously reported [49]. Briefly, heart specimens were hybridized with digoxigenin-labelled anti-sense riboprobes of CVB3, followed by incubation with anti-digoxigenin antibody conjugated to alkaline phosphatase (Roche, #11093274810). Vector Red substrate (Vector Laboratories, #SK-5100) was used for color detection of viral RNA.

### Real-time quantitative RT-PCR

Total RNA was extracted from rat neonatal cardiomyocytes and cDNA was synthesized as previously described [8]. Transcript levels of *CryAB* were measured by the TaqMan technology (Life Technologies, #4331182) using CryAB probe (Rn01421541_m1) on a ViiA 7 Real-Time PCR system (Applied Biosystems) and normalized to *GAPDH* (Rn01476455_m1) mRNA following the manufacturer’s instruction.

### Statistical analysis

Results are presented as means ± standard deviation (SD). All data presented are representative of at least three independent experiments. Statistical analysis was performed with unpaired Student’s t-test. A p value of less than 0.05 was considered to be statistically significant.

## Abbreviations

PQC: protein quality control
CryAB: alphaB-crystallin
CVB3: coxsackievirus B3
DCM: dilated cardiomyopathy
sHSP: small heat shock protein
PAO: pre-amyloid oligomers
H&E: hematoxylin & eosin
WT: wild-type
SD: standard deviation
